# A randomized clinical trial comparing internal and external pessaries in the treatment of pelvic organ prolapse in postmenopausal women: A pilot study

**DOI:** 10.1016/j.clinsp.2024.100335

**Published:** 2024-03-13

**Authors:** Renato Sugahara Hosoume, Thais Villela Peterson, José Maria Soares Júnior, Edmund Chada Baracat, Jorge Milhem Haddad

**Affiliations:** Faculdade de Medicina, Universidade de São Paulo, São Paulo, SP, Brazil

**Keywords:** Pelvic organ prolapse, Pessary, Quality of life, Randomized controlled trial

## Abstract

**Introduction and hypothesis:**

Internal vaginal pessary is among the leading treatments for pelvic organ prolapse (POP); however, it has a high adverse event rate. An external pessary was recently developed as an alternative. The study's objective was to compare the efficacy of external and internal pessaries in treating POP in postmenopausal women.

**Methods:**

This parallel randomized (1:1 ratio) open-blind study included 40 symptomatic women with stage 2 or 3 POP. They were randomized into two groups: group 1 (internal pessary) and group 2 (external pessary) (*n* = 20 in each); and evaluated at the start of and 3 months after the treatment. Statistical analysis was performed to compare the results within and between the groups before and after the 3-month treatment.

**Results:**

The groups were homogeneous, except for the variables previous pregnancies (*p* = 0.030) and POP-Q score of apical prolapse (*p* = 0.023) whose values were higher in group 2. A significant improvement in quality of life was observed in both groups after 3 months of follow-up; however, internal pessaries were found to be more effective (*p* < 0.001). In group 1 there were differences between the initial and final POP-Q scores of anterior (0.004) and apical prolapse (*p* = 0.005). The complication rate associated with internal pessary use was high (*p* = 0.044).

**Conclusions:**

The present data suggested that external pessaries have a similar effect to internal ones for the treatment of POP and improvement of the quality of life of postmenopausal women.

## Highlights

• Internal and external pessaries improved the quality of life of women with pelvic organ prolapse.
• The use of an internal pessary changed the POP-Q stage related to prolapse of the anterior and apical vaginal compartments in women with pelvic organ prolapse.
• Studies investigating alternative treatments for Pelvic organ prolapse, such as the use of an external pessary, are extremely important.

## Introduction

Pelvic Organ Prolapse (POP) is defined as the descent of pelvic organs through the vagina [1]. This is a common condition in women, and it can be detected during physical examination in 40% to 60% of multiparous women [2]. POP significantly affects a patient's life and their body image, habitual activities, sexual function, and quality of life [3].

Treatment can be either surgical or conservative, the latter including physical therapy through the strengthening of the pelvic floor muscles and vaginal pessary use. Surgical treatment is indicated for women with POP symptoms who had no success with conservative treatment, but reoperation rates can reach 10.5% [4]. With regard to conservative treatment, a pessary is a silicone device inserted vaginally that can be used as an alternative to surgery in the clinical management of POP. Its main advantages are its low cost and high acceptance [3,5,6], despite high rates of adverse events, which can reach 32% [7].

**Fig. 1:**
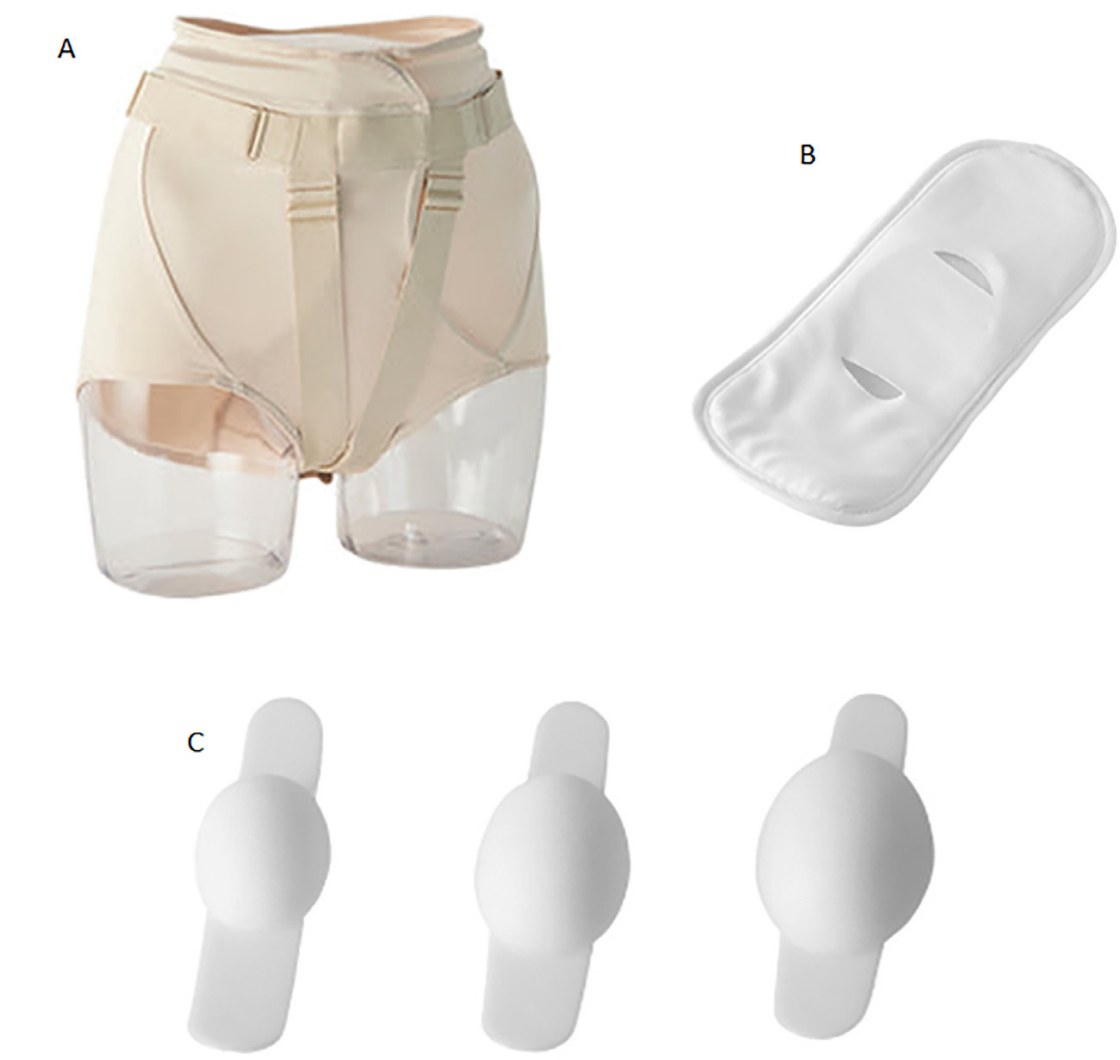
Components of the external pessary. Figure2A. adjustable panty-shaped support; Figura2B. tampon-like holder; Figure2C silicone cushion.

External pessaries have recently been developed as an alternative to traditional pessaries, which are used internally in the vagina. These new devices are composed of three parts: an adjustable panty-shaped support (Fig. 1A), a tampon-like holder (Fig. 1B), and a silicone cushion (Fig. 1C). The silicone cushion supports the prolapsed organs, and it comes in three sizes, which is chosen according to the size of the vaginal opening and POP. The holder absorbs urine and secretions, and it is used to lock the cushion in place and prevent it from being displaced. The adjustable support encloses the holder and the cushion.

Because external pessaries are not placed inside the vagina and are removed daily for hygiene, they may be associated with a lower risk of complications, such as vaginal discharge, bleeding, vaginal erosion, migration to other pelvic organs, and incarceration, compared with internal pessaries [7]. Internal pessaries have a 49% discontinuation rate [7] and can cause adverse events in up to 32% of women who use them [8]. Although adverse events are usually mild, neglecting the pessary can cause severe complications such as urogenital fistulas and migration to the abdominal cavity.

The surgical procedures have high rates of recidiva and some women with decompensated disease are not suitable for surgery. Thus, studies investigating alternative treatments for POP, such as the use of an external pessary, are extremely important. This hypothesis is that the external pessary is effective similar to the internal one for treatment and to improve the quality of life of postmenopausal women with POP.

## Materials and methods

### Study design, recruitment, and inclusion and exclusion criteria

This study was approved by the Ethics Committee of the University Clinics Hospital of the Faculty of Medicine of the Universidade de São Paulo under n° 80,899,517,200,000,068.

This was a parallel controlled randomized study with two arms and a 1:1 allocation ratio. Women with POP stage 2 and 3 symptoms according to the Pelvic Organ Prolapse Quantification (POP-Q) classification [9] who accepted to participate in the study were included. All women were patients seen at the University Clinics Hospital of the Faculty of Medicine of the University of São Paulo, Brazil, from July 2018 to May 2019. The women were divided into two groups: one group of patients receiving an external pessary and one group of patients receiving an internal pessary [10].

In fact, the inclusion criteria are post-menopause women with pelvic organ prolapse stage 2 and 3 according to the POP-Q who agreed to participate in the study.

The exclusion criteria were neoplasias of the genitourinary tract, postmenopausal genital bleeding, repeat urinary infection, vaginal stenosis, short vagina, repeat genital infection, impossibility of follow-up or adequate pessary maintenance, and contraindications to estriol use. The women were followed up for 3 months after the insertion of the vaginal pessary.

### Intervention

After signing the consent form, the participating women were assigned to one of the groups (external or internal pessary) according to a randomization performed at the https://www.sealedenvelope.com website. The use of 0.5 g of estriol vaginal cream (1 mg/g) twice a week during treatment was prescribed to all patients.

The internal pessary used was produced by the company Medical Software e Equipamentos Médicos (São Leopoldo, State of Rio Grande do Sul, Brazil). The model used was the ring without a silicone membrane. After the patient's initial evaluation and randomization to the internal pessary group, vaginal length and width were measured, the adequate pessary size was estimated from these measurements, and the device was then inserted into the vagina. Subsequently, the patients were asked to perform the Valsalva maneuver or to cough. The pessary was considered to be properly inserted if it was not expelled and did not cause discomfort. To evaluate the patient's comfort in using the pessary, she was asked to walk, squat, and sit down. Finally, the patient was asked to urinate, to check for obstructive symptoms [11,12]. The women who used internal pessaries were instructed to remove it once a week for cleaning with water and neutral soap and reintroduce it afterward [12].

FemiCushion™ brand external pessaries were used (Women's Medical Research, Inc., Tokyo, Japan). Three sizes are available for the adjustable support, which are chosen according to abdominal circumference measurement. The pessary was considered to be properly inserted if it did not cause discomfort. The patients who used external pessaries were instructed to remove them every night before bed to maintain the hygiene of the device and adjustable support.

Sociodemographic data (age, parity) and clinical data (comorbidities, prolapse symptoms, previous surgeries) were collected before the start of the treatment. Additionally, the symptoms and quality of life of all the patients were evaluated using questionnaires during the initial visit and at the end of the study (after 3 months). Validated Brazilian Portuguese translations of the following questionnaires were used: Prolapse Quality of Life Questionnaire (PQOL) [13], Pelvic Floor Bother Questionnaire (PFBQ) [14] and Pelvic Floor Distress Inventory (PFDI) [15]. The prolapse was classified according to the Pelvic Organ Prolapse Quantification (POP-Q) system [9] by an experienced gynecologist before and 3 months after treatment (immediately after the device was removed).

The women from both groups were re-evaluated one week after the initial visit, 15 days after the second visit and monthly for 2 months, for a total of four visits. A thorough gynecological examination was performed during the follow-up consultations to check for possible complications related to the use of the pessary.

At the end of the third month, the patients were again interviewed and examined, and they answered the questionnaires for quality of life, evaluation of symptoms, and satisfaction with the treatment used. POP-Q and treatment complications were evaluated once more.

The subjective cure criterion was determined based on the answer to question 2 of the PQOL questionnaire: ‘How much do you think your prolapse problem affects your life?’ When the answer was ‘It does not affect my life at all’ at the final three-month re-evaluation, a subjective cure was considered to have happened [13,16]. The objective cure criterion established for the study was a POP-Q score ≤ 0 at the re-evaluation at 3 months after treatment.

### Statistical methods

No similar study was done in the literature, which impairs sample size calculation. It is a pilot study. The qualitative variables were expressed as absolute and relative frequencies. With regard to the quantitative variables, the mean, median, first quartile (Q1), third quartile (Q3), minimum and maximum value, and standard deviation were calculated.

Comparisons between independent groups were evaluated using the Mann-Whitney non-parametric test. The Wilcoxon non-parametric test was used to compare dependent groups. The association between the qualitative variables was evaluated using either Pearson's Chi-square test or Fisher's exact test. The McNemar test was used to evaluate dependent groups (qualitative variables).

An intention-to-treat analysis was performed. The significance level adopted was 5% for all hypothesis tests. All statistical analyses were performed using the SPSS statistics software (IBM Corporation, Armonk, NY, USA), version 25 for Windows.

## Results

Forty women were included and evaluated in this study. The patients were randomized electronically into two groups ‒ 20 women in the internal pessary group and 20 women in the external 156 pessary group.

When the initial data was evaluated, no significant difference was observed between the two groups in terms of clinical and sociodemographic characteristics, with the exception of the number of previous pregnancies, which was higher in the external pessary group (*p* = 0.030) (Table 1). Additionally, the groups were similar in terms of the POP-Q classification of cystocele and rectocele (*p* > 0.05). The initial POP-Q classification of apical prolapse showed a significant difference between the two groups, with the external pessary group presenting a more pronounced apical prolapse (*p* = 0.023) (Table 2). The evaluation of POP-Q points Ba, Bp and C before treatment revealed no significant differences between the groups, both quantitatively (mean and median) and qualitatively (prolapse point > 0) (*p* > 0.05) (Table 2).

**Table 1:** Epidemiological and clinical characteristics of the patients who participated in the study.

Group		External	Internal	p-value
		*n* = 20	*n* = 20	
Age (years)	Mean (SD)	68.2 (9.3)	68.6 (12.4)	0.901^a^
	Median (min–max)	68.4 (47.8–82.3)	69.6 (35.5–83.3)	
Number of pregnancies	Mean (SD)	6.3 (4.1)	3.5 (2.4)	0.030^b^
	Median (min–max)	4.5 (2–14)	3 (0–9)	
Number of vaginal deliveries	Mean (SD)	5.2 (3.9)	3.1 (2.3)	0.121^b^
	Median (min–max)	3.5 (1–12)	3 (0–9)	
Number of caesarean sections	Mean (SD)	1 (0.6)	0.3 (0.6)	0.314^b^
	Median (min–max)	0 (0–2)	0 (0–2)	
Number of interrupted pregnancies	Mean (SD)	1 (0.9)	0.1 (0.5)	0.068^b^
	Median (min–max)	0 (0–3)	0 (0–2)	
Type II diabetes mellitus	Yes	5 (25.0%)	6 (30.0%)	0.723^c^
	No	15 (75.0%)	14 (70.0%)	
Systemic arterial hypertension	Yes	13 (65.0%)	12 (60.0%)	0.744^c^
	No	7 (35.0%)	8 (40.0%)	
Menopause	Yes	20 (100%)	19 (95.0%)	_1_^d^
	No	0	1 (5.0%)	
Previous surgeries	Yes	8 (40.0%)	8 (40.0%)	_1_^c^
	No	12 (60.0%)	12 (60.0%)	
Previous hysterectomy	Yes	5 (25.0%)	4 (20.0%)	_1_^d^
	No	15 (75.0%)	16 (80.0%)	

SD, Standard Deviation; min, minimum value; max, maximum value.

a Student's *t*-test.

b Mann-Whitney test.

c Pearson's Chi-Square test.

d Fisher's exact test.

**Table 2:** POP-Q staging and evaluation of POP-Q points, Ba, Bp and C, according to the treatment groups (external and internal) before the treatment.

Measurement	Group			p-value
	External	Internal	Total	
	*n* = 20	*n* = 20	*n* = 40	
	n (%)	n (%)	n (%)	
*Cystocele POP-Q classification*				0.131^a^
No prolapse	0	3 (15.0)	3 (7.5)	
Stage 1	1 (5.0)	1 (5.0)	2 (5.0)	
Stage 2	3 (15.0)	6 (30.0)	9 (22.5)	
Stage 3	16 (80.0)	10 (50.0)	26 (65.0)	
*Rectocele POP-Q classification*				0.720^a^
No prolapse	2 (10.0)	3 (15.0)	5 (12.5)	
Stage 1	2 (10.0)	3 (15.0)	5 (12.5)	
Stage 2	5 (25.0)	7 (35.0)	12 (30.0)	
Stage 3	11 (55.0)	7 (35.0)	18 (45.0)	
*Apical prolapse POP-Q classification*				0.023^a^
No prolapse	1 (5.0)	0	1 (2.5)	
Stage 1	8 (40.0)	10 (50.0)	18 (45.0)	
Stage 2	0	5 (25.5)	5 (12.5)	
Stage 3	11 (55.0)	5 (25.5)	16 (40.0)	
*POP-Q Ba (cystocele)* (>0)	17 (85.0)	13 (65.0)	30 (75.0)	0.144^c^
(≤0)	3 (15.0)	7 (35.0)	10 (25.0)	
Mean (SD)	2.4 (1.7)	1.1 (2.5)	1.7 (2.2)	0.106^b^
Median (Q1–Q3)	2.5 (2.0; 3.0)	1.5 (−0.5; 3.0)	2.0 (0.25; 3.0)	
*POP-Q Bp (rectocele)* (>0)	12 (60.0)	8 (40.0)	20 (50.0)	0.206^c^
(≤0)	8 (40.0)	12 (60.0)	20 (50.0)	
Mean (SD)	1.4 (2.2)	0.3 (2.6)	0.9 (2.5)	0.115^b^
Median (Q1–Q3)	2.0 (0; 3.0)	0 (−2.0; 2.0)	0.5 (−1.0; 3.0)	
*POP-Q C (apical prolapse)* (>0)	11 (55.0)	6 (30.0)	17 (42.5)	0.110^c^
(≤0)	9 (45.0)	14 (70.0)	23 (57.5)	
Mean (SD)	0.1 (4.3)	−1.2 (3.8)	−0.6 (4.1)	0.392^b^
Median (Q1–Q3)	2.5 (−4.5; 4.0)	−1.5 (−4.0; 1.5)	−0.5 (−4.0; 3.0)	

POP-Q, Pelvic Organ Prolapse Quantification; SD, Standard Deviation; Q1, First Quartile; Q3, Third Quartile.

a Fisher's exact test.

b Mann-Whitney test.

c Pearson's Chi-Square test.

**Fig. 2:**
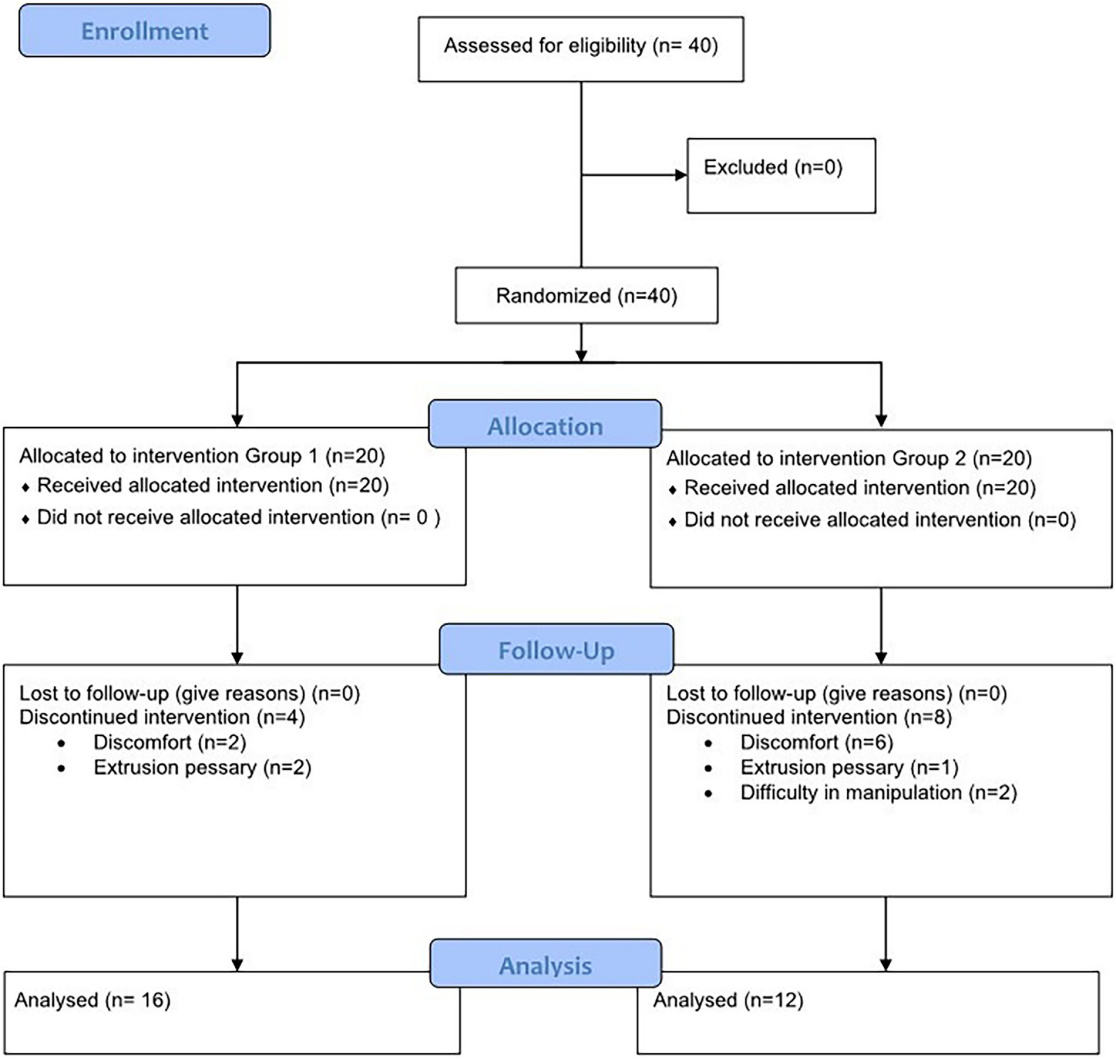
Flow chart (CONSORT diagram).

During the 3-month follow-up, 12 patients discontinued the treatment (4 from the internal pessary group 1 and 8 from the external pessary group). In the internal pessary group, two patients discontinued the treatment due to discomfort and two due to device displacement during the first week of follow-up. In the external pessary group, six patients discontinued the treatment due to discomfort, one due to difficulty in using the device, and one due to device displacement during the first week of follow-up. At 3 months after treatment, a total of 16 and 12 patients of the internal and external pessary groups, respectively, were re-evaluated (Fig. 2). The treatment discontinuation rate in 3 months was similar for both groups (*p* = 0.168).

The quality-of-life questionnaire scores of women with POP were analyzed according to the type of pessary used. Significant differences were observed between the initial and final PFBQ scores of women of both groups. Additionally, there were differences in the PFDI and PQOL score distributions. The differences between the groups were also tested for each of the evaluations (initial and final), and no differences were found between the groups in terms of the quality-of-life questionnaire scores (Table 3).

**Table 3:** Initial and final evaluations of the PFBQ, PFDI and PQOL scores according to the type of pessary used in the patients who participated in the study.

		Initial evaluation	Final evaluation	p-value^a^
		*n* = 40	*n* = 28	
**Total PFBQ score**				
*External*	Mean (SD)	35.11 (28.68)	11.85 (19.26)	0.003
	Median (Q1–Q3)	26.67 (10.00–54.44)	2.22 (0–14.44)	
*Internal*	Mean (SD)	39.56 (20.64)	7.64 (10.13)	<0.001
	Median (Q1–Q3)	38.89 (17.78–55.56)	2.22 (1.11–12.22)	
p-value^b^		0.297	0.962	
**Total PFDI score**				
*External*	Mean (SD)	82.40 (63.18)	24.83 (42.61)	0.006
	Median (Q1–Q3)	70.31 (35.42–129.17)	8.33 (0–19.79)	
*Internal*	Mean (SD)	80.42 (44.70)	19.21 (26.07)	0.001
	Median (Q1–Q3)	85.42 (37.50–101.04)	6.25 (0–36.46)	
p-value^b^		0.685	0.924	
**Total PQOL score**				
*External*	Mean (SD)	35.77 (23.50)	12.19 (16.36)	0.005
	Median (Q1–Q2)	31.99 (17.65–51.47)	7.72 (1.10–10.29)	
*Internal*	Mean (SD)	32.83 (18.65)	4.64 (8.49)	0.001
	Median (Q1–Q2)	32.72 (16.54–46.32)	1.84 (0.37–4.41)	
p-value^b^		0.695	0.084	

PFDI, Pelvic Floor Distress Inventory; PFBQ, Pelvic Floor Bother Questionnaire; PQOL, Prolapse Quality of Life Questionnaire; SD, Standard Deviation; Q1, First Quartile; Q3, Third Quartile.

a Wilcoxon test for dependent samples.

b Mann-Whitney test.

With regard to subjective cure, 75% of the women in the internal pessary group were cured, whereas 15% of the women in the external pessary group were cured (*p* < 0.001).

Differences between the initial and final evaluations of POP-Q points Ba and C were statistically significant in the internal pessary group (*p* < 0.05). No differences were found between the initial and final evaluations of points Ba, C, and Bp in the external pessary group (*p* > 0.05) (Table 4).

**Table 4:** POPQ classification according to the type of pessary used in the patients who participated in the study before and after the treatment.

		Evaluation		
		Initial (*n* = 40)	Final (*n* = 28)	p-value^a^
**External (POPQ)**				
*BA Cystocele*	Mean (SD)	2.4 (1.7)	1.8 (1.9)	1
	Median (Q1-Q3)	2.5 (2.0; 3.0)	2.0 (0.5; 3.0)	
*BP rectocele*	Mean (SD)	1.4 (2.2)	1.2 (2.2)	0.317
	Median (Q1-Q3)	2.0 (0; 3.0)	2.5 (0; 3.0)	
*C Apical Prolapse*	Mean (SD)	0.1 (4.3)	−0.2 (3.9)	1
	Median (Q1-Q3)	2.5 (−4.5; 4.0)	0 (−3.5; 3.0)	
**Internal (POPQ)**				
*BA Cystocele*	Mean (SD)	1.1 (2.5)	−0.7 (1.7)	0.004
	Median (Q1-Q3)	1.5 (−0.5; 3.0)	−1.0 (−2.0; 0.5)	
*BP rectocele*	Mean (SD)	0.3 (2.6)	−0.6 (1.6)	0.165
	Median (Q1-Q3)	0 (−2.0; 2.0)	0 (−1.5; 0)	
*C Apical Prolapse*	Mean (SD)	−1.2 (3.8)	−3.1 (2.7)	0.005
	Median (Q1-Q3)	−1.5 (−4.0; 1.5)	−3.5 (−4.0; −2.5)	

POP-Q, Pelvic Organ Prolapse Quantification; SD, Standard Deviation; Q1, First Quartile; Q3, Third Quartile.

a Wilcoxon test for dependent samples.

The objective cure rate was found to be higher in the internal pessary group than in the external pessary group when the POP-Q points Ba (cystocele) (*p* = 0.003), Bp (rectocele) (*p* = 0.011) and C (apical prolapse) (*p* = 0.004) were evaluated. This was also observed in the evaluation of all the points combined (*p* = 0.006).

Complications were evaluated according to the type of pessary used. Significant differences were observed between the two groups, with a high incidence of complications in the internal pessary group (*p* = 0.004) (Table 5).

**Table 5:** Occurrence of complications in the study participants.

	Type of pessary		
	External	Internal	p-value
	*n* = 20	*n* = 20	
	n (%)	n (%)	
**Did the patient have complications?**			0.044^a^
Yes	1 (5.0)	7 (35.0)	
No	19 (95.0)	13 (65.0)	
**Which complication did the patient have?**	*n* = 1	*n* = 7	
Vaginal discharge	0	4 (57.1)	0.106^a^
Pessary displacement	1 (100)	3 (42.9)	

a Fisher's exact test.

## Discussion

Pelvic organ prolapse is a common condition in women [2]. POP significantly affects patient's quality of life [3]. Choosing the perfect treatment can be a challenge and may be either surgical or conservative. Surgical treatment is indicated for women with POP symptoms who had no success with conservative treatment, but reoperation rates can reach 10.5% and can be contraindicated according to the clinical conditions of the patient [4]. With regard to conservative treatment, the internal pessary can be used as an alternative to surgery in the clinical management of POP but has high rates of adverse events, which can reach 32% [7]. This study presented comparative results obtained after 3 months of treatment with either internal or external pessary provided to women with POP up to stage 3. In the present study, external pessary has a similar effect to internal pessary for treatment of POP and improvement of the quality of life of postmenopausal women.

The analysis of the quality of life of the study participants revealed significantly improved scores in all domains of the Quality-Of-Life questionnaires (PQOL, PFDI, and PFBQ) for both the groups analyzed separately before and 3 months after treatment. With regard to subjective cure, which was considered to have happened when the question “How much do you think your prolapse problem affects your life?” was answered with ‘It does not affect my life at all’ in the final re-evaluation, 75% of the women in the internal pessary group and 15% of the women in the external pessary group were subjectively cured.

With regard to external pessary, the only study found in the literature that evaluated the relationship between this type of device and quality of life included only five women with POP who used the device for 3 months. The quality of life of four of those women improved [17]. Although it was a small study, its result is in line with the present findings.

The analysis of the association between internal pessary uses and quality of life improvement revealed results that were similar to those found in the literature. A systematic review showed that this type of device improves the quality of life of women with POP. The authors concluded that internal pessaries improve the quality-of-life scores because they reduce both the urinary and the intestinal symptoms associated with prolapse [7].

Another study evaluated the effect of the use of internal pessaries on the quality of life of 97 women with stage 3 or 4 POP and concluded that these devices had a positive impact on women's quality of life. Additionally, the use of the pessary had a 90.7% efficacy rate and high satisfaction rates (75.3%) [6].

In their study, Mao et al. included 142 women with POP who were treated with vaginal ring pessaries and had a mean follow-up time of 17 months. Their quality of life before and after the treatment was evaluated using validated questionnaires. It was also concluded that pessaries are a safe option for treating POP as they significantly improved the women's quality of life, and there were no serious adverse events [18].

The results of the present study showed that both external and internal pessaries improve the quality of life of women with POP. However, internal pessaries, which are already used in the treatment of POP, offer higher subjective cure rates when the goal is complete improvement of the discomfort caused by POP [19].

A significant response in the POP-Q classification of the anterior and apical compartments was observed in the internal pessary group when comparing the classifications performed before and 3-months after treatment. However, this response was not observed in this group's posterior compartment. No difference was observed in the POP-Q classification of any compartment in the external pessary group when comparing the classifications performed before and 3 months after treatment.

The comparison of the objective cure rate of the groups, considering a POP-Q score ≤0, revealed a higher cure rate among women in the internal pessary group than among those in the external pessary group for all the POP-Q points evaluated. The findings described above showed that internal pessaries were superior in treating POP when the POP-Q stage was evaluated.

No studies can be found in the literature evaluating the relationship between external pessary use and POP-Q staging. External pessaries act as external supports for the prolapsed organs and are not inserted in the patient's vagina. This may explain why no changes in terms of POP-Q staging were observed in the women who used this type of device.

The results described above for the internal pessary are in line with those of some studies published in the literature. The study by Mendes et al. included 50 women with POP who were treated with internal pessaries and re-evaluated after 4 months of treatment and showed a reduction in prolapse according to the POP-Q classification 72 h after pessary removal [20].

A report of a case series with six women with uterine prolapse who used a pessary as treatment for a mean time of 27 months, which was removed at this time, and were followed for a mean time of 42 months after pessary removal, showed complete prolapse reduction [21]. Another study evaluated 19 women with POP who were treated with an internal pessary for one year and re-evaluated after that period and showed that the POP-Q stage had regressed when a re-evaluation was performed 48 h after the device was removed [22].

In the present study, the evaluation of POP-Q stage 3 months after treatment was performed immediately after the pessary was removed from the vagina. A longer interval between pessary removal and clinical evaluation would be more adequate; however, in clinical practice, women resist being without the pessary for long periods, for fear of prolapse recurrence.

The studies that evaluated the POP-Q stage in women with POP treated with pessaries also included a re-evaluation performed a short time before pessary removal. Thus, it is possible that POP-Q stage improvement does not persist in the long term in women with POP treated with internal pessaries that are later removed [20,21].

The complication rate in the external pessary group was 5%, with the only complication being pessary displacement and in the internal pessary group was 40%; five cases (25%) were described as vaginal discharge, and pessary displacement was reported in three other cases (15%). Some studies evaluated the complications of internal pessaries, which ranged from 56% to 58%. The main complications reported were: pessary displacement (28%), bleeding (6%–6.8%) and vaginal discharge (22%–26%); these results are similar to those obtained in the present study 23, 24, 25.

Another study, published in 2017, evaluated 140 women diagnosed with POP who were treated with an internal pessary and showed a rate of pessary displacement of 26% and a rate of vaginal discharge of 17%; these rates are similar to those obtained in the present study [26]. No serious complication, such as urogenital fistula, vaginal cancer, or displacement of the device into the abdominal cavity was reported in the present study. However, these complications are usually associated with neglected pessaries [27,28].

It is of note that, although no statistically significant difference was observed between the two groups in the occurrence of vaginal discharge alone, a difference was observed in the clinical practice, i.e., no women in the external pessary group had vaginal discharge compared to 5 (25%) women in the internal pessary group. This is a common adverse event in women with POP who use internal pessaries, and it can affect up to 17% of them [26]. Thus, external pessaries can be considered an alternative POP treatment, particularly for women who use an internal pessary and have vaginal discharge very frequently.

The main strength of the present study was its originality, as there is no study in the literature investigating the efficacy of external pessaries in the treatment of POP. Another strength was the fact that the authors performed a randomized study. The limitation of this study was the short follow-up period. In addition, the number of women who discontinued study participation before the end of the treatment is another important issue.

## Conclusion

The present data suggested that the external pessary is a similar effect to the internal one for the treatment of POP and improvement of the quality of life of postmenopausal women. Furthermore, complication rates were higher in women who used an internal pessary; however, all the complications were minor.

## Authors’ contributions

RS Hosoume: Conceptualization, Data curation, Formal analysis, Investigation, Methodology, Project administration, Writing - original draft, Writing - review & editing. TV Peterson: Conceptualization, Data curation, Formal analysis, Writing - original draft. JMS Junior: Writing - review & editing, Supervision. EC Baracat: Writing - review & editing, Supervision. JM Haddad: Conceptualization, Data curation, Formal analysis, Methodology, Project administration, Writing - original draft, Writing - review & editing, Supervision.

## Funding

This research did not receive any specific grant from funding agencies in the public, commercial, or not-for-profit sectors.

## Prior presentation

None

## Declaration of competing interest

The authors declare no conflicts of interest.
